# Crisis and Sensemaking: The Relevance of Liminal Experience and Metastability for a Sociocultural Psychology of Crisis

**DOI:** 10.1007/s12124-025-09943-2

**Published:** 2025-10-23

**Authors:** Paul Stenner

**Affiliations:** School of Psychology and Counselling, The Open University, Milton Keynes, UK

**Keywords:** Liminality, Crisis, Disaster, Equilibrium, Metastability

## Abstract

This paper provides an overview of liminality theory and its relevance to the study of sensemaking in and about crises. It sketches a model of liminal metastability as a way of thinking about disrupted equilibrium within psychosocial systems. Liminal metastability presupposes a reality composed of a complex array of forms of process, and hence calls for a relational process ontology. In developing the concept of liminal metastability, the paper discusses and critiques Weick’s account of sensemaking in crisis. It also discusses the accounts of disturbed equilibrium at play in cognitive dissonance theory and Freud’s theory of disruptions to the pleasure and reality principles.

## Introduction

The process thinker A.N. Whitehead (1911, p.61) identified social progress, not with *more thinking*, but with an extension of the number of operations which can be performed *unthinkingly*. Thoughtful operations, he suggested, ‘are like cavalry charges in a battle — they are strictly limited in number, they require fresh horses, and must only be made at decisive moments’. But he did not undervalue thinking on account of its rarity. On the contrary, he recognised that a ‘general idea… is always a danger to the existing order’ (Whitehead, 1933, p.17). This is because thoughtful abstractions are potent weapons for controlling our thought about more concrete facts. In what follows I argue that the concepts of liminality, liminal experience and liminal metastability are useful transdisciplinary theoretical resources for a sociocultural psychology of crisis grounded in process thought. Liminality theory can help us to think systemically yet processually from the micro to the macro and across physical, organic, psychological and societal levels (Stenner, 2022).

My aim in what follows is to show how liminality theory can aid the development of a socio-cultural psychology of crisis with transdisciplinary potential. At the heart of this will be a concept of *disrupted liminal metastability* that builds on existing accounts of equilibrium and its loss in various types and levels of ‘system’. This will require some critical engagement with systems theory, which is currently the predominant integrative and transdisciplinary scientific framework, and which usually works with a concept of equilibrium or homeostasis. This critique gives importance to the precise nature of the ‘subject-in-crisis’ (i.e. the one whose equilibrium is compromised by crisis). Although much systems theory remains mechanistic and non-processual, we will see how the concept of autopoiesis, informed by second-order cybernetics, makes sensemaking core to reflection on crises, and pushes towards a liminality-theoretic orientation. Also, from a historical perspective, Freudian theory can be viewed as adding new complexity by taking seriously Fechner’s notion of a psychic system (always embedded in social systems) proper to the ‘subject in crisis’. Freud in turn influenced Festinger’s cognitive dissonance theory, a classic social psychological theory born out of reflection on crisis. From dissonance theory arose the work of Karl Weick, one of today’s leading theorists of crisis and sense-making. Together, I try to show how these traditions for studying sensemaking in crisis build towards a concept of liminal metastability that moves beyond more mechanistic notions of disrupted equilibria. They build towards a processual sociocultural psychology capable of showing how crises disrupt the liminal metastability of becomings at multiple and interconnected ‘system’ levels (the physical, biological, psychological and sociological) from the microscopic to the planetary.

## Initial Overview of a Liminality-Theoretic Approach Embedded Within a Relational Process Ontology

Liminality is a term coined by Victor Turner in response to Arnold van Gennep’s identification of a liminal phase within rites of passage. It is an abstract concept which points to the possibility of liminal experience as a distinctive mode of experience. Liminal experience is relevant to crises because it is associated with occasions of significant psychosocial transformation (Stenner and Moreno, 2013, Stenner & Zittoun, 2020). It is ‘psychosocial’ because, in managing situations in which people ‘transition’ between different forms of societal order, it facilitates the integration of subjectivity into fields of collective organisation. As a synthetic concept, liminality also has potential to unify and integrate research across the sciences and it is this potential I wish to explore.

In my book *Liminality and experience* (Stenner, 2017a) I draw two fuzzy theoretical distinctions (which function as contrasts). First I distinguish ‘liminal experience’ from the more usual and familiar ‘pivotal’ or ‘canonical’ experiences of so-called ordinary, structured, workaday life settings. Pivotal experience is associated with forms of activity that tend to be regularised such that we can, to return to Whitehead, perform important operations without thinking too much about them. The psychosocial transformations associated with liminal experience, by contrast, tend to be occasioned by or during transitions in which the forms and relations which previously structured experience and conduct are either (a) deliberately suspended (as in the transition phase of a ‘rite of passage’ through which people pass from one of life’s ‘stations’ to another) or (b) disrupted, ruptured or otherwise interrupted by some unexpected event (Stenner, 2017a). Thus having distinguished liminal from pivotal experience, I roughly distinguish these two types of liminal experience, calling a) *devised* liminality and b) *spontaneous* liminality (the first often layered upon the second to facilitate subjective transformation suited to transit between different organised settings).

Liminal experience thus presupposes the prior existence of a *structured form of social order* that is, perhaps temporarily, suspended, lost, ruptured, interrupted. I actually prefer Whitehead’s expression *forms of process* because this accentuates how the ‘material’ that is formed, deformed and reformed is itself complex and processual in nature. As Whitehead puts it:

Nothing is more interesting to watch than the emotional disturbance produced by any unusual disturbance of the forms of process. The slow drift is accepted. But when for human experience quick changes arrive, human nature passes into hysteria. For example, gales, thunderstorms, earthquakes, revolutions in social habits, violent illnesses, destructive fires, battles, are all occasions of special excitement. There are perfectly good reasons for this energetic reaction to quick change. My point is the exhibition of our emotional reactions to the dominance of lawful order, and to the breakdown of such order. When fundamental change arrives, sometimes heaven dawns, sometimes hell yawns open (Whitehead, 1938, p. 95).

But liminality is not the sheer negative collapse of order. It is not chaos, but the suspension of prior order: one is no longer what one was (in the prior social form), but not yet what one will become (in the next form of social process). In this respect liminal experience is not purely negative (not purely a loss of powers) but *ambivalent* and *paradoxical.* For example, those going through the devised liminal phase of an initiation rite are framed within a symbolism for which they are *no longer* children (subject to the norms structuring social relations involving children) but *not yet* adults (subject to newly appropriate duties and expectations). This temporal hiatus can be generalised in a deeply paradoxical form expressing that those going through liminal occasions of becoming are existentially ‘*both* both/and *and* neither/nor’ (Kofoed & Stenner, 2017). Liminal occasions can, but also need not, open potentials which lead to the creation of new forms of process necessary for productive change and development (i.e. pattern shift). In short, ‘pivotal’ psychosocial forms of order are structured in large part by shared mutual expectations (which make coordinated social activity possible), and liminality arises when, for whatever reason, these expectations no longer hold, need to be exchanged for others, or have become unsustainable.

In human societies, ‘social structure’ (formed process) mediated by expectations arises from the routinisation of preferred ways of actualising potentials at play in an environment, and since liminality involves the ‘release’ of these potentials, it can be construed as an occasion of ‘potentialisation’ (Andersen & Stenner, 2020, Stenner & Andersen, 2020, Andersen et al., 2025). Any process of structural re-generation is thus likely to involve a renewal or extension of ‘sensemaking’ that has been disturbed, such that new expectations arise that are capable of making use of lost potentials previously sacrificed to structure (Andersen & Stenner, 2024). ‘Cultural experience’ (Winnicott, 1971, Zittoun & Stenner, 2021), and particularly ‘aesthetic experience’ becomes decisively important in this process of renewal because ‘devised liminal experiences’ (mediated, for example, by aesthetic forms) amplify imagination and creativity. Obviously, from a research perspective, it is crucial to understand the nature of the forms of process that have been disrupted and the complex processes through which liminal experience might become productive rather than purely destructive (Bjergkilde & Stenner, 2021). These are quite tricky theoretical tasks, rarely broached. The point is that unlimited potentiality can be hugely destructive and un-liveable because - not being ordered into proper and predictable spaces and times via shared expectations - it comes thick and fast (everything, everywhere, all the time, always). Finding value in this ‘blooming, buzzing confusion’ should not be taken lightly.

The distinction between pivotal and liminal experience might map onto different modalities of sensemaking. Crudely, crises can pull pivotal experience (including conduct and sensemaking) in a liminal direction, and ‘cultural experiences’ (e.g. those devised experiences afforded by painting, art, literature, music, etc) can facilitate creative sensemaking following spontaneous ruptures (Stenner & Zittoun, 2020; Zittoun & Stenner, 2021, Wagoner & Zittoun, 2021). This should encourage attention to the role of cultural experience (and aesthetic experience in particular) in facilitating sensemaking that can be helpful, not just with coping, but with cultivating forms of becoming that enable learning in the wake of crises.

The concept of a ‘liminal hotspot’ should be briefly noted in this overview (Stenner et al. 2017). Experiences of transformation are delicate matters and this is reflected in the very existence of cultural means for scaffolding, supporting and, importantly, tolerating them. A liminal hotspot is a stuck transition in which transformations become problematic for those involved. Ambiguous, ambivalent and paradoxical experiences are typical of liminal occasions precisely because the shared norms and mutual expectations structuring pivotal experience no longer hold. When liminal experiences are deliberately devised (as in ritual), these unusual experiences can be fostered and put to use in generating, for example, an unusually intense sense of communitas amongst the ‘liminars’. But spontaneous loss of structure can lead to liminoid experiences lacking temporal or spatial containment and which unfold without tolerance from others or transformative relevance to self and community. Instead of going through a personally and collectively recognised becoming, people can be held suspended in these liminal hotspots, neither what they were nor what they might become. In previous work my colleagues and I have tried to delineate how sensemaking is affected under such circumstances of stuck transition. Where paradox is generated but not tolerated, for instance, there is a tendency for sensemaking either to *paralyse* conduct (because no logic for action can be settled upon) or to *polarise* by ‘flattening’ the paradox (allowing action based on ignoring the ‘contradictory’ aspects of the actual experience, which thus come back to ‘bite’). Escape from a liminal hotspot requires sensemaking which creates new forms capable of more productively de-paradoxifying these challenging impasses. Liminal hostpots too can vary in scale from the minimicro to the macro: Might world society today, with its ‘disaster capitalism’ and its global self-definition in terms of ‘times of crisis’ or ‘catastrophic times’, be conceived as a liminal hotspot?

A final point to note in this overview is that liminality theory foregrounds *situated sensemaking* as a core *subject matter* of sociocultural psychology. Sensemaking is the set of processes through which we make sense of things. Sensemaking is not just the core subject matter providing sociocultural psychologists with something to study, it is also what sociocultural psychologists do. Our activity can be well described as *second-order sensemaking*, because we ‘make sense’, in as disciplined (empirical and theoretical) a way as possible, of the sensemaking of others around us. Societal self-descriptions can also be considered second-order sensemaking. How does today’s global society make sense of itself? I hazard, for example, that it no longer defines its leading edge as ‘modern industrial society’ or ‘postmodern post-industrial society’, but as ‘society *in crisis*’. What are the implications of this self-description?

## Systems Theory and the Notion of Crisis as Disturbed Equilibrium

Since the early 20th century, systems theory has been the leading force urging for and practicing transdisciplinarity. Systems theory is unavoidable given our concerns. It introduces a concern with sensemaking in the context of systemic thinking and foregrounds the idea that crisis entails the disruption of an equilibrium state proper to a system. The notion of second-order sensemaking introduced above (and also used by Weick, 2022) comes from von Foerster’s (1970) systems-theoretical argument for a second-order cybernetics capable of recognising that the cybernetic subject matter being observed is also an observer (see Stenner, 2004 and Brown & Stenner, 2009). Von Foerster notes that a ‘cybernetic’ question arises whenever one deals with a system in which *effectors* (an engine, some muscles, etc.) are connected to and guided by *sensory organs* in a circular organization (Dewey, 1896, had already laid the ground for this in his reformulation of the concept of the ‘reflex arc’). Sensemaking in this cybernetic sense is first of all prospective or future facing, but this requires some capacity for memory (if only repeating a simple programme).

When approaching what is at play in the important systemic notion of a disrupted state of equilibrium we must be able to specify the *subject* whose state of equilibrium might be disrupted or threatened with disruption (i.e. the equilibrium of *what* or *who*, and *from whose perspective*?). We must also recognise that ‘crisis’ is usually a designation offered by a sensemaking observer, and hence an attribution whose circumstances and content deserve critical attention in themselves (Rosen et al., 2023).

For general systems theory the *subject* whose state of equilibrium might be disrupted by crisis is assumed to be a *system* of some kind. This premise follows from the transdisciplinary view that nature (‘the universe’) itself is a system of systems of various kinds and scales. This means that ‘the system’ in crisis can be lots of things, and this variation cannot be taken lightly. The system may be physical and mechanical (a computer is a system, as is our solar system) or it may be a living cell (an organic system). It may also be an entire individual organism, like a fly composed of a society of living cells and tissues whose equilibrium is threatened by the tongue of a frog. Or it may be an entire societal organisation composed of relations between people and things, like a school judged to be in crisis by the body assigned to evaluate it (in the UK context, that second-order system is called Ofted). In the case of the recent pandemic, the system serving as subject-of-crisis might be called ‘global society’ as such. For the environmental crisis the ‘subject’ at issue is broader still, because planet earth is construed as an integrated system of processes who equilibrium is disrupted. Obviously the larger the scale of the system envisaged the greater the scope for a crisis to affect everything subsumed as a ‘component’ (or better, *relatum*) under that system. Also a crisis might spread ‘bottom up’ from a ‘location’ in a sub-system of a larger system as when the SARS-CoV-2 virus mutated in a market in Wuhan and entered human cells locally before spreading Covid-19 across the globe and transforming practically every social system on earth (Andersen et al., 2025). So to grasp how a crisis affects the equilibrium of a system we must grasp the nature (the forms of process) of that system: A virus is a system, a cell affected by a virus is a system, a body composed of cells is a system, an organisation composed of relations between acting bodies is a system, cybernetic theory is a system of scientific propositions, etc.

This kind of systems-theoretical terminology and conception is far from new and – since it was first proposed by Von Bertalanffy in 1928 - some form of it tends to be generally accepted across the sciences, whether explicitly or implicitly. General systems theory remains relevant but requires some reformulation. From a liminality-theoretic perspective some of these core concepts take on a distinctive meaning (see Stenner, 2005, Andersen et al., 2025). First and foremost, ‘system’ should be written with inverted commas because a ‘system’, of whatever kind, is, as already hinted, actually a complex of interrelated ‘forms of process’, and hence a formation in process of incompletion. In my own work I have drawn upon the philosophy of A.N. Whitehead (who actually was an inspiration to Von Bertalanffy) to ground this process-ontological view of systems (Stenner, 2008, 2017a), but Michel Serres (1982, 73) puts it extremely well when he writes:

Maybe there is or never was a system … The only instances or systems are black boxes. When we do not understand, when we defer our knowledge to a later date, when the thing is too complex for the means at hand, when we put everything in a temporary black box, we prejudge the existence of a system. When we finally open the box, we see that it works like a space of transformation. The only systems, instances, and substances come from our lack of knowledge. The system is non knowledge. The other side of nonknowledge. One side of nonknowledge is chaos; the other, system. Knowledge forms a bridge between the two banks. Knowledge as such is a space of transformation.

Systems theoreticians can be more or less ‘process ontological’ in their thinking and hence more or less sensitive to the relevance of liminality theory. Leaving aside for now the process through which ‘crisis’ is attributed to a system, whatever type or scale of system is under consideration, any ‘equilibrium’ subject to interruption must be reckoned as the temporary result of a process or set of processes. Indeed, in ‘crisis’ the ongoing nature of the constitutive processes becomes newly visible because it can no longer be taken for granted *by* the system. Accordingly, in a crisis, the system must *do something different* or else risk a transition from development to decadence (in the case of social systems) and perhaps death (in the case of a living system).

### The Concept of Autopoiesis in Systems Theory

There is a second sense in which there is no ‘system’. There is no system without relationality to an environment which is itself composed of ‘systems’ of various kinds. This means that there is no such thing as an isolated ‘system’. Within systems theory this point is often grasped in terms of the Bergsonian distinction between ‘open’ and ‘closed’ systems, but a leap beyond this conception was attained in biology with Maturana and Varela’s concept of *autopoietic* (self-producing) systems (see Andersen & Stenner, 2023, Andersen et al., 2025). From this perspective a system (whether a cell or a complete organism) becomes self-producing only by *closing itself off* from its environment such that, as far as its own operations are concerned, it is self-referential (see Stenner, 2004, 2005). Of course, each cell and each organism still absolutely depends upon its environment and so must be ‘open’ to that environment (a living system, for example, requires food to get the energy it needs to reproduce itself from its own elements), but the insight of autopoietic systems theory is that any such openness is managed as a function of *operational closure* (operations are processes, and so autopoietic systems theory takes a step closer to a process ontology when it affirms that *operations* are the basic elements of a system, and a step closer to liminality theory when it emphasises the semi-permeable membrane, threshold, skin, border or boundary).

When, for example, the system eats, or if the system ‘observes’, it does so by self-reference to its own internal operations, closed off by a permeable boundary. It is, in short, the system itself which must create and ongoingly re-enact the distinction between itself and its environment. Since each system must distinguish itself from its environment, each one creates an environment that is distinctly its own. Compared to other more mechanical forms of systems theory (but in line with von Foerester), autopoietic theory also makes the notion of the system’s own sensemaking (‘self-reference’) fundamental. The fact that the system is itself an observer (an experiencer and sensemaker), makes subjectivity and communication essential to these forms of systems theory that are ripe for convergence with liminality theory. Again, it also becomes important to be able specify *who* is the observer in situations of crisis (is it a crisis *for* the system, or for another system busy making second-order observations?).

The operational closure of a self-producing (autopoietic) system means that it relates to its environment mostly, as it were, indirectly, i.e. by means of its relations to its own recursive operations. But if it is to reproduce itself through time it’s internal operations must allow it some purchase on its environment, without which it would swiftly perish. Animals, for example, gain what might be called their *sense of reality* in large part (though perhaps not exclusively) through internal operations associated with their senses. This is poorly understood as a matter of the environment somehow directly entering the system through the senses (information-based cognitive theories were based on this false start). Actually the senses merely provide ways for the organism to feel the responses of particularly sensitive parts of its own body as it encounters possible things-of-interest. The visual system of a frog is attuned to detect small dark moving objects which when detected rapidly unleash its long sticky tongue (these sensory and motor systems being tightly coupled into a single sub-system). The frog is less ‘interested’ in the internal complexity of the fly-qua-system (to which it has no access or direct connection) because the ‘fly’ is objectified *for* the frog’s experience *by* and *in* that ‘experience’ (and, of course, with reference to the possibility of eating that fly via the motor response of a tongue-dart).

Nevertheless, this indirect and mediated connection (e.g. between frog and fly) is sufficiently *congruent* (a word used this way by James, 1987, p. 746 before Maturana) to allow the frog-qua-system to navigate and manage its environment according to its needs. The reason for this improbable success is that system and environment (an environment always including other systems), have evolved together over a long time. The frog does not need to learn about ‘flies’ because this relationship is built into the structure of its sensory-motor system (although perhaps its system may be fine-tuned through the ontogenesis of a given frog). This is essentially what Maturana called *structural coupling*: the structure of both organism and medium are broadly congruent because they change together through time. Structural coupling means that system and environment, system and other systems, become mutually adapted over time as they evolve, develop and act together. For example, people develop from the dependent babies they were into the adults they become via a history of gradual transformation in which their milieu changes together with them. Very crudely, we can’t become a jet pilot or bus driver without a sustained process of learning how to fly jets or drive buses, and without jets and buses in the environment obviously there are no jet pilots. When structurally coupled, the structures of organism and medium change in concert, together.

This processual reformulation of the concept of system brings us to the problem of equilibrium which in my view also needs to be reformulated into a concept of *liminal metastability* if it is to help sensemaking about crises (Stenner, 2021). Crudely, liminal metastability is associated with the phase in between moments of structural *de-coupling* and *re-coupling.*

## The Notions of Equilibrium (1865) and Homeostasis (1926) in Organic Living Systems

Equilibrium can be simply understood as something that is *conserved* by means of structural coupling. Maturana gives the simple example of skiing. The ever changing and finely adjusted movements of a skier as they slide down a snowy slope are designed to conserve their equilibrium as they descend in that very specific mountain environment. They stay sliding upright by constantly making tiny adjustments to suit the ever-changing circumstances they face as they descend. Hence system and medium or environment constantly change together but in a manner that conserves equilibrium. None of this could happen without the sense of reality gained through educating the senses and educated sensemaking. The ski novice constantly loses that equilibrium and falls, but as they learn the skill they get a more finely tuned sense of the challenges facing them and their responses to them. What applies to skiing also applies, not just to walking and talking, but to living as such. At the risk of being corny, we are thrown onto the ski slope of life and we slide until we finally fail to conserve our equilibrium. The way we slide is partly dictated by the slope, but also according to the path picked out thanks to what is conserved by our sensemaking. So our drift is more or less modified by responses afforded by our sensemaking (always in concert with the actions of our bodies).

Cybernetic/systems theoretical accounts of equilibrium built upon developments in biology that shed new light on living systems. Claude Bernard systematically applied the idea of equilibrium to physiology in 1865. He introduced the notion of a ‘milieu intérieur,’ the constancy of which must be conserved amidst changes in the ‘milieu extérieur’. Sixty years later Walter Cannon coined the term homeostasis to generalise Bernard’s notion. As Cannon (1926, p.91) put it:

The constant conditions which are maintained in the body might be termed equilibria. That word, however, has come to have fairly exact meaning as applied to relatively simple physico-chemical states, in closed systems, where known forces are balanced. The coordinated physiological processes which maintain most of the steady states in the organism are so complex and so peculiar to living beings - involving, as they may, the brain and nerves, the heart, lungs, kidneys and spleen, all working cooperatively - that I have suggested a special designation for these states, homeostasis. The word does not imply something set and immobile, a stagnation. It means a condition - a condition which may vary, but which is relatively constant.

Equilibrium and homeostasis thus both concern the ongoing structural coupling of system and environment in which ‘conditions’ are ‘conserved’ amidst change (i.e. remain relatively constant despite environmental variation). The internal structures of a system function to conserve the ‘equilibrium’ (of that system’s ongoing autopoietic processes of self-production) in relation to structures discerned in its environment. If ‘biological’ equilibrium is lost (e.g. the interior milieu exceeds or falls below its threshold temperature, or lacks water or energy), a crisis ensues and death swiftly follows. ‘Psychosocial’ equilibrium, however, is slightly more complex, and theory must be adjusted accordingly. Again, to broach this issue we must recognise it as a matter of specifying the nature of the *subject of crisis* (the nature of the system entering crisis). The flows of process at play in different system types vary from flows of energy in ‘physical’ systems, to self-catalysing biochemical reactions in ‘organic’ systems, to concatenated streams of experience in ‘psychic’ systems, to the to-and-fro of communicative interchange in ‘social’ systems, and of course the *ongoing relations* between *all* these flows become ever more relevant as systems ascend in terms of the complexity or degree of abstraction of their main operative medium (see Stenner, 2017a).

### From Fechner To the Freudian Equilibrium of Thanatos

Just a few years earlier than Cannon, Freud (1920) extended the notion of biological equilibrium to psychic systems in a quite dramatic way in his *Beyond the pleasure principle*. In his previous work Freud had essentially worked with a theory of pleasure derived from Gustave Fechner. Freud states quite clearly that his ‘pleasure principle’ is merely ‘a special case under Fechner’s principle of the “tendency towards stability”’ (277). Fechner’s ‘principle of constancy’ basically equates pleasure with stability and unpleasure with loss of stability, and this equation was what Freud had taken forward. To follow Freud’s pleasure principle is thus simply to aim towards keeping the quantity of ‘free excitation’ (as opposed to ‘bound excitation’) within the psychic system *constant*, or at least as low as circumstances permit. Pleasure = reducing free excitation and keeping it within stable bounds. Under normal circumstances the pleasure principle and the reality principle work together in this task. The reality principle is a modification of the pleasure principle that occurs when the instinctive ‘animal’ operating in its libidinal medium proves capable of paying heed to the civilised communicative demands of others. Together ‘pleasure’ and ‘reality’ principles conspire to pleasurable stability, even if sometimes we use circumstances of play, dream or masochism (all dealt with by Freud) to surreptitiously snatch some (equally pleasure/stability oriented) ‘wish-fulfilment’ in the darkness behind the back of our super-ego.

In his 1920 essay, however, Freud (1920) criticises his own prior (Fechnerian) assumptions about the psychic relevance of ‘pleasure’ (the pleasure/stability equation). This is based on his observations of situations in which people seem to *seek* displeasure. For obvious reasons these situations seemed to him ‘beyond’ the pleasure principle that had guided his prior theoretical efforts. They go against the basic motivational assumption of the stability-seeking union of pleasure and reality principle. Freud starts to see, for example, that supposedly pleasurable activities like play can focus on displeasure: ‘in their play children repeat everything that has made a great impression on them in real life’ … ‘If the doctor looks down a child’s throat or carries out some small operation on him, we can be quite sure that these frightening experiences will be the subject of the next game’. It is as if there is a modality of sensemaking – associated with trauma and crisis - in which the usual directive to seek pleasurable stabilisation were somehow reversed. It is as if a shocking event can dislodge sensemaking from the logic of the pleasure principle itself, and open it onto a new, mysterious kind of logic. A shocking event can ‘provoke a disturbance analogous to a traumatic neurosis; and only after the binding has been accomplished would it be possible for the dominance of the pleasure principle (and of its modification, the reality principle) to proceed unhindered’ (Freud, 1920).

In this essay Freud implicitly entertains two different explanations of these phenomena ‘beyond’ the logic of the pleasure/stability equation. He fixates on the more glamourous of the two explanations, and virtually ignores the more productive. It is the more productive explanation (which we will examine a little later), that will lead us to a concept of liminal metastability proper to the complexity of human psychosocial systems and to the nature of their responses to crisis (Stenner, 2021: 182–187).

The more glamorous explanation invoked the figure of *Thanatos* and ‘explained’ the repetition of displeasure as a ‘compulsion’ to repeat which expresses a ‘daemonic’ will to death. Freud repeatedly uses the word daemonic to describe the various ‘compulsions to repeat’ (e.g. in the play of children post-trauma) that concern him in the essay. He was under the impressive influence of the second law of thermodynamics. The first law expresses the *conservation* of energy as it shifts forms (i.e. the scientific basis of Fechner’s constancy principle) while the second ‘entropic’ law states a trajectory of systems towards thermodynamic equilibrium. The second law is the ‘daemon’ silently leading all forms of matter to their heat death. In thrall of Thanatos, Freud even proposes subsuming pleasure itself under the influence of the death drive: ‘The pleasure principle seems actually to serve the death instincts’ (Freud, 1920, p. 338). Fechner’s definition of pleasure as restoration of a prior state of equilibrium gave Freud a scientific basis for associating ultimate stability with the inanimate inertness of death. Lacking a better concept of equilibrium, Freud thus takes the thermodynamic equilibrium of the second law as the standard governing all natural equilibria. But in fact, stable equilibrium, if we follow thermodynamic principles, is a negative extreme and not a standard: it belongs only to that which has exhausted the dialogue between novelty and repetition. Stable equilibrium ‘excludes the idea of becoming because it corresponds to the lowest level of potential energy possible; it is the sort of equilibrium that is attained in a system when all the possible transformations have been achieved and no other force remains to enact any further changes’ (Simondon, 2020).

As already outlined above, the equilibrium of a living organism is never in fact stable in this manner, but a *form of processes* structurally coupled to its environment such that it has (temporarily) achieved the regular, habitual, canonical consistency characterising what I have called ‘pivotal experience’. Autopoietic equilibrium must in fact be tilted or inclined such that its stability is never in fact inert or static (one is reminded of the clinamen of Lucretius). A corollary to this is that strictly speaking homeo*stasis* must always *flow* and hence, Serres somewhere suggests, is better named homeo*rhesis*. In both cases (equilibrium and homeostasis), the form of stability/constancy at play can be better grasped as *metastability*, the interruption of which is complex and, as it were, thought (and consciousness) provoking.

## From Freud To Simondon: From Pivotal To Liminal Metastability

Gilbert Simondon’s (2020) distinction between stable equilibrium and metastability is seminal here (see Stenner, 2021, Wrbouschek & Slunecko, 2021). Metastability implies the difference between the potential and the actual. It is a condition in which potentialities hover on the brink of becoming actual. Metastability is not unique to living systems. Even a crystal ‘individuates’ via a physical process through which the metastable chemical potentiality of a supersaturated solution is progressively actualised (Simondon, 2020). But it becomes unavoidable with the emergence of life. A living system, being negentropic, must maintain its own systemic continuity through a constant actualisation of newly generated potentials, and it must constantly regenerate its own source of potentiality. This is why food is a defining feature of whatever lives. When, for example, a living system eats from its environment (or photosynthesises its ‘food’ if a producer), it is ongoingly renewing an internal supply of metastability by extracting from its food the potential energy needed for the self-perpetuation of its operations. With any living system the balance between repetitive and novel ways of actualising metastable potentials is skewed in the direction of novelty. With the further evolution of human mentality and sociality—mediated by layers of symbolic reference carried through experience with ‘culture’— this balance shifts even further towards novelty. In human culturally mediated life, for example, some of the metastable potentials at play can be consciously entertained as alternative possibilities for action and articulated in a discursive proposition (even if that alternative is a proposition like ‘distrust innovation and return to tradition’).

So, when we meditate on the idea that crises follow (or perhaps predict) disruptions to equilibrium states it is important to take careful stock of the fact that the nature of this ‘equilibrium’ depends upon the nature of the system affected (crystal? Jellyfish? Human family?). When considering a crisis affecting some human life, for example, we must recognise that this life entails a hugely complex web of structural coupling between interacting persons. Each person in that web, qua biological system, has evolved phylogenetically to be maximally flexible to ontogenetic refinements of individual development (the evolution in humans of a large and massively plastic brain capable of significant developmental change, not to mention learning, in early life, for example). But also, that ontogenetic development unfolds within and is scaffolded by the sociogenesis of an historically ‘evolved’ social order with a ‘culture’ whose symbolism resonates with the psychic structure of each emergent ‘self’ (See Brown et al., 2025). Our experienced human ‘worlds’ are the complex symbolically mediated products of this web of structural coupling such that our very selves are also the outcome as much as the origin of our (collective) ‘sensemaking’. Freud, more than most, was aware of this complexity, as is expressed in his complex model of the psyche. Aware that the human subject spans biology (organic forms of processes), psychology (experiential processes) and sociology (communicative processes), he envisaged the human subject as itself internally divided, with the ‘id’ representing animal biological instincts, the ‘super-ego’ representing the observed requirements of social order, and the poor ‘ego’ left hanging over the unconscious ravine between the two bickering forces.

Given this complexity (involving structural couplings of multiple forms of process), it is clear why it is that the metastability of our human ‘worlds’ is tightly connected with our capacities for sensemaking (including symbolically mediated communication). This is the source of the second and more productive explanation of the ‘beyond’ phenomena that Freud ultimately sacrificed to Thanatos. Observing children who ‘repeat’ negative experiences in play, Freud also suggested that this might allow the child to ‘pass… over from the passivity of the experience to the activity of the game’ or, put slightly differently, to ‘abreact the strength of the impression and, as one might put it, make themselves master of the situation’ (p. 286). This is rather different from the suggestion of compulsive repetition driven by an unconscious will to death. Rather, thrown off-kilter by a trauma, shock or crisis, play allowed these children to take stock of what happened, make new sense of it, and get back in the driving seat, perhaps virtually at first. Using this mode of explanation, ‘children repeat unpleasurable experiences’ not because of daemonic compulsion, but because ‘they can master a powerful impression far more thoroughly by being active than they could by merely experiencing it passively’. In liminality-theoretic terms, they are separated from their prior complex metastability by a spontaneous de-stablilising event, they go through a phase of liminal metastability (in which the spontaneous experience is accompanied by techniques for devising related experiences in more controllable ways), and, ideally, this liminal process creates a path for ‘reincorporation’ into a renewed phase of pivotal metastability. And in this, Freud is surely correct (and prescient) to note, albeit implicitly, the commonalities between the liminal cultural spheres of play, dream, art, theatre and therapy.

Freud, in this second mode of explanation, correctly draws us to contemplate motivations beyond the pleasure and reality principles. Pleasure and reality principle-oriented sensemaking, viewed this way, is *pivotal* sensemaking. It makes sense when *we* make sense (i.e. to ourselves) and when *our world* makes sense. As such, it presupposes the metastability born of coherent and successfully congruent webs of structural coupling. In crisis situations it is possible that what once was coupled becomes uncoupled, incongruous, uncanny. Self-interested rationality is all very well when we know what our self *is* and what its interests *are*, but if we enter a phase of liminal metastability we may lack exactly this. Certainly a person ‘repeating’ (the inverted commas are necessary because in fact there is no exact repetition) a disturbing experience in play or dream or art or literature or therapy (each a ‘world within a world’) may feel as if they were somehow possessed by a force beyond their comprehension, since there are indeed unconscious forces at play here that escape the usual pleasure and reality principle-oriented self-interested rationality. But the daemon is not in fact a positive force, but a distorted way of making sense of the loss of structure actually at play in the circumstances Freud concerned himself with. From this liminality-theoretical perspective, the pleasure principle and its beyond express the contrast between two different modes of sensemaking proper to liminal and pivotal experiences: it is the liminal experience of being thrown out of one’s pivotal world and into a situation ‘between worlds’ that opens ambivalent possibilities beyond the pleasure principle. The repetitions of the Beyond need not spell death but more life and indeed *more than* life. The possibility of the existence of liminal experience enters because human psychosocial existence is such a complex composition of movement that it can be moved in its movement and transformed in its mode of transformation. Liminal experience is experience *of* such movement and transformation. To paraphrase Whitehead, when it happens, hell’s very jaws can seem to open, yet at the very same time we may get a glimpse of something like heaven.

### The Role of Liminal Affective Technologies in Steering Liminal Metastability

In sum, if I am correct then the phenomena so astutely identified by Freud in 1920 can be understood as liminal features proper to a metastable system that has been thrown beyond the normative structure of its usual ‘working equilibrium’. And this means that, without naivety, we should not neglect the positive potentials of the ‘beyond’ (with its media of play, art, theatre, therapy) as a site for the regeneration of sensemaking in the wake of crisis. We should not forget that Nietzsche, who influenced Freud, also contrasted the equilibrium state of the thermodynamics of his time with his preferred philosophy of eternal return. For him, equilibrium was nothing but a resting place for the inertly identical, whilst ‘eternal return’ signified the being of that which perpetually becomes. And it was Nietzsche who drew new attention to the cultural historical relevance of the birth of tragedy in creating a theatrical space/time of possibilities serving as a crucible for mediating such becomings. Also, Freud’s love of Sophocles and Shakespeare was arguably the true motor of psychoanalysis (just as Vygotsky’s grasp of Hamlet was the inspiration for his psychology). And after Freud, Winnicott gave us his ‘third space/time’ which he envisaged as a ‘potential space’ (composed of a dance of structural coupling between carer and infant) necessary for the individuation of an infant’s ‘unit self’. For Winnicott, all art and culture springs from this curious field of indistinction between self and environment to which we can and perhaps must ever return for creative sustenance. And after Winnicott, Victor Turner, writing in turn of liminal rites, endlessly circled around the need to describe and stress the import of an ephemeral subjunctive quality of ‘trembling in the balance’ (Turner, 1982, p. 44). Turner gave us the concept of anti-structure, and he drew upon Sutton-Smith’s theory of human play as proto-structural: a ‘latent system of potential alternatives’ which precedes and exceeds what he calls the ‘working equilibrium … of normative structure’. Turner too, insisted upon emphasising the hidden relevance not just of ritual, but also of theatre, and he picked up on Nietzsche’s hints concerning the historical emergence of theatre from ritual (see Stenner, 2017b).

An entire genealogy should be written about the cultural mediation of liminality, with genealogy itself included as the preferred method of scholars of liminality (Szakolczai, 2023). We see a thread of continuity linking ‘media’ of play, ritual, theatre, etc. as ‘liminal affective technologies’ which summon and foster ‘as if’ worlds within worlds which feed an insatiable forward thrust into the future, opening new ways of creating and resolving the paradoxes of metastability (Zittoun & Stenner, 2021; Andersen & Stenner, 2020). If each of these thinkers is associated with moments of transition or great change, it is because the liminal experience that preoccupies them is experience of structural *un*coupling (the suspension or collapse of forms of order) so that more adequate structural couplings can, perhaps, be discovered.

## Cognitive Dissonance, Crisis and Liminality

We now turn to the more familiar social psychology of crisis. Weick’s first publication in 1964 was on the reduction of cognitive dissonance through task enhancement and effort expenditure, so before turning to Weick I will sketch how dissonance theory (which had its hay-days in the 1960s) fits my argument. This tradition began by staging problems like: how do people resolve the uneasy feeling that might follow when two incompatible experiences are simultaneously entertained? For example, if a person chooses to undertake the action of giving a public talk expressing a viewpoint contrary to their actual opinion, they may resolve any felt ‘dissonance’ by bringing their opinion into closer alignment with what they said. Festinger’s approach, inspired by Lewin, enabled a focus on sensemaking. We might say that participants in dissonance experiments ‘make sense’ of the uncomfortably dissonant feeling by finding a way of removing it. Sensemaking here is equivalent to finding ways to render inconsistencies more consistent.

As was fashionable in those days, Festinger presented his theory as thoroughly counter-intuitive (as if *inconsistent* with common-sense). But arguably all these insights are already present in Aesop’s fable of the sour grapes. The fabled fox can’t reach the delicious looking grapes, and so decides they must be sour. It doesn’t take away or add much to suggest that the fox resolved the dissonance of its uncomfortable predicament by changing its attitude towards the grapes. Either way, with Aesop we have a giggle at the cunning fox’s brazen inconsistency. So whilst dissonance theory is not against common-sense, it did run against the orthodoxy of the behaviourist tradition still dominant during the 1950s. Festinger and Carlsmith (1959), for example, found that the less participants were paid (in a task they had cleverly designed to generate dissonance), the more they say they like this task. Superficially at least, a behaviourist view would say that the more rewarded an action is, the more liked it should become.

Festinger, like Lewin, rejected the behaviourist vision of a subject passively driven by reward contingencies and both set up their experiments to depict social behaviour as the conduct of a sensemaking organism preoccupied with the *consistency* of their experience. Dissonance theory is inconsistent with behaviourism, but in this respect it is notably consistent with psychoanalysis. Festinger (1957, p.2) happily uses the term *rationalization* to designate the way our own inconsistencies rarely show up as such to us (we otherwise so cunning foxes like to think ourselves consistent). Indeed, he opts to use the musical word ‘dissonance’ rather than the more logical sounding ‘inconsistency’ to draw attention to the *psychological discomfort* felt when different portions of an experience fail, as it were, to harmonize into ‘consonance’. Two hypotheses arise from this perspective: that folk are motivated to reduce the discomfort of dissonant inconsistency to more comfortable consonant consistency; that experiences predicted to create or add dissonance will be avoided. Put more simply: approach consonance, avoid dissonance. Festinger is perfectly happy to assert that exactly the same would apply should the word ‘dissonance’ be replaced with ‘hunger’, ‘frustration’ or ‘disequilibrium’. (p.3). We are motivated to reduce hunger by eating, to reduce frustration by overcoming the obstacle to our desire and to reduce disequilibrium by restoring equilibrium. All could be described as ‘dissonant’ (hunger and frustration are dissonant feelings, and equilibrium is restored by eating or removing the obstacle frustrating satisfaction).

Festinger makes clear that it was speculation about responses to disasters that led him to dissonance theory. He had data (reported by Prasad, but uncited) on rumours that spread following the so-called ‘Great Indian earthquake’ which struck the Bihar-Nepal region in 1934, killing over 10,000 people. Most of these rumours, Festinger reports, predicted worse quakes soon to come, and these were widely accepted. Why spread and accept anxiety provoking rumours? Festinger’s answer was that perhaps they weren’t anxiety producing but anxiety *justifying.* Perhaps the rumours helped people make sense of the dissonance that may have resulted from the fact of being scared yet without any continuing justification: ‘Perhaps these rumours provided people with information that fit with the way they already felt’ (Festinger, 1957, p. vii). Festinger’s insight was that people seek information that fits with how they are currently reacting. The scary rumours fit with the fear they already felt.

It should be clear that Festinger, probably via Heider’s balance theory, was still very much working the same territory as Freud. Instead of Fechner’s notion of pleasure as the reduction of unpleasurable disequilibrium we have the motive to reduce the dis-pleasurable inconsistency now called dissonance. Also, just as Freud wondered why children would repeat negative experiences in their play, Festinger wonders why people in earthquake situations would repeat (and lap-up) negative rumours about worse disasters to come. Freud was speculating beyond the pleasure principle of Fechner’s psychophysics, Festinger was experimenting beyond the pleasure principle of Watson’s behaviourism. In my reading, both were actually running up against the difference between sensemaking proper to pivotal and to liminal modes of experience.

Above I twice summed up dissonance as arising from inconsistency amongst *experiences* (or aspects of experience). In fact, this is already me ‘appropriating’ the theory. Festinger and his followers had little stomach for talk of ‘experiences’ and preferred the much narrower and more scientific-sounding term *cognitions*. For example he defines dissonance as ‘the existence of nonfitting relations among cognitions’ (p.3). This might seem pedantic but I think it has important implications. There can be nonfitting (i.e. incongruent) relations between two beliefs, but Festinger also often writes about inconsistencies between cognition and behaviour. But this is not inconsistent given his definition of cognition as ‘any knowledge, opinion, or belief about the environment, about oneself, or about one’s behaviour’ (3). So if a given belief is inconsistent with a ‘behaviour’, then we have ‘nonfitting relations among’ these two *cognitions* (we must be cognisant of our behaviour, and cognisant also our belief). Does this suggest that we must be cognisant also of the discrepancy between them? Well, this additional step is not stipulated in the above definition, where dissonance simply is the *existence* of these ‘nonfitting relations’. And yet Festinger is happy to use the term ‘rationalization’ to designate situations in which nonfitting relations exist but are NOT recognised by the subject. So it seems dissonance is not simply the existence of nonfitting relations between cognitions but presupposes also awareness of that kind of relation. Three cognitions are thus needed to issue in the feeling of dissonance (e.g. a cognition about a behaviour, a cognition about a belief, and a cognition about the incompatibility of these two).

This ‘triple cognition’ entails a lot of complicated abstract mediation. In the name of parsimony, is it not more simple to say that different aspects of our *experience* are felt as dissonant? Surely ‘experience’ is a more inclusive concept than cognition (cognition being a rarefied experience oriented to knowledge). If sufficiently reflexive we can experience our own behaviour, our own cognition, our own beliefs and we can also experience a sense of disquiet (or not) when these do not cohere congruently. It is interesting to ask whether anything is lost or gained by suggesting also the phrase ‘experience of liminality’ in place of ‘cognitive dissonance’. Actually, from my perspective, Festinger’s emphasis on cognition expresses the superficiality of pivotal not liminal experience. In liminal experience we *go through an experience* and are *moved* by it, incongruence and all. In pivotal experience we are not stirred to our depths but simply register things that are happening as if the events were purely external, each neatly ‘cognised’ with its own proper name. During pivotal experience, the metastability of our adequate structural coupling allows us to more or less accurately and thoughtlessly predict what will happen next and expect it. Pivotal experience has become such because it is *structured by workable expectations* (and it is structured both by and for the experiencing subject and for others who communicate and form ‘social systems’ with that subject). Of course we experience all kinds of little blips, worries and noises, but these can happily be edited out and made to disappear. Our experience and conduct, in this way, are regularised, rationalised and justified. ‘Cognitive dissonance’ is a name for such blips, worries and noises, such faintly disturbing ‘inconsistencies’ which our pivotal sensemaking is well equipped to edit and iron-out into a workable semblance of *consistency.* Pivotal experience has plenty of contacts to fetch it facts to get it back on track when something attacks its imagination.

But after a certain threshold of disruption, these little dissonant experiences, these easily managed micro-liminalities, can accumulate into a rather different ‘liminal’ mode of sensemaking for which their noise is transformed into signal and inconsistency may be welcomed as contrast and paradox. Instead of massaging inconsistencies in the medium of cognition into more agreeable forms, during liminal experience the agreeable forms themselves melt, de-form and de-differentiate. We enter liminal metastability.

During pivotal experience it is as if our sensemaking had a centripetal dynamic which pulled any anomalous aspects of experience towards the normative centre of a seemingly coherent field characterised by a semblance of self-contained subjectivity. During liminal experience, by contrast, sensemaking acquires a centrifugal dynamic which pulls towards the peripheral borders of the field which is characterised by ambiguity, paradox, incoherence and ambivalence. These liminal features are proper to a subjectivity in process of becoming other than itself (not least because the periphery of any field is a threshold which necessarily blurs and shades into neighbouring fields). On the one side, the superficial (cognitive) aspects of dissonant experiences are resolved into the coherence of pivotal metastability, on the other, that surface is swept aside by stirrings from the affective depths of a nascent subject in process of emergence. If the latter subject might actually prove capable of acknowledging the contradictions that the former was condemned repeatedly to deny, then we might have a productive development: a pattern shift (Greco & Stenner, 2017). This would mean that pattern shift requires a phase of liminal metastability.

## Karl Weick on Sensemaking in Crises

Karl Weick work has been seminal to social psychological research into why sensemaking is relevant to crises (Weick, 1995). It is ripe for development in the direction we are suggesting, having moved ever closer to a process ontology, albeit avoiding the passage of liminality theory (contrast Weick, 1991 with Weick, 2022). Can we make sense of this particular sensemaking tradition from a liminality perspective? Weick’s is an interesting case of an experimental social psychologist who, after becoming a theorist of organisations (a management guru even), shifted towards mostly qualitative and theoretical studies. Informed by the broadly ‘constructionist’ and sociological psychology associated with Garfinkel, Goffman, Sacks and others, Weick embraced the notions of performativity and enactment at play in the constructionist micro-sociology that had thrived outside of mainstream experimental social psychology. The influence of Garfinkel’s ethnomethodology on Weick’s (1991, p.106) definition of sensemaking is obvious when he writes that his focus on sensemaking ‘emphasises that people try to make things rationally accountable to themselves and others’ and that ‘the basic idea of sensemaking is that reality is an ongoing accomplishment that emerges from efforts to create order and make retrospective sense of what occurs’.

Weick’s use of this concept of sensemaking in the study of crises and disasters began with a 1988 paper entitled ‘enacted sensemaking in crisis situations’. He was concerned to address the human element in crises that were then being approached in terms of technological failures, but to go beyond the focus on decision-making (Maitless and Sonnenshein, 2010). Defining crises as ‘low probability/high consequence events’, he observes that crises put sensemaking under particular strain. At the best of times our actions often run ahead of our understanding of them, but in crises the ‘retrospective’ nature of sensemaking is amplified. Unexpected events produce large effects and one action, retrospectively shown to be misjudged, can escalate disastrously. This gives particular importance to initial responses to a crisis, since these shape the crisis trajectory. Weick’s research *modus operandi* is to reanalyse existing analyses of cases of crisis or disaster and to add further illumination with a focus on how sensemaking contributed to the crisis situation. In the 1988 article, Weick re-analysed Shrivastava’s (1987) analysis of an event during which more than 2000 people died from a poison gas leak at a Union Carbide plant in Bhopal, India, in 1984. The proximal cause of the toxic leak was the failure to insert a slip blind after removing (to clean) a pipe connected to a methyl isocyanate (MIC) tank, but Weick traces this and other lapses to a broadly shared managerial assumption about the relative unimportance of the Bhopal plant. He notes a self-fulfilling prophesy whereby a ‘plant perceived as unimportant proceeds to act out, through turnover, sloppy procedures, inattention to details, and lower standards, the prophecy implied in top management’s expectations’ (Weick, 1988: 313).

Two more examples provide some ground for our liminality theoretic re-evaluation of Weick’s work.

### The Mann Gulch Fire Crisis

A 1993 article deals with the Mann Gulch fire disaster that Killed 13 men in Montana in 1949 and that had been the subject of a 1992 book by Norman Maclean. As with Bhopal, Weick (1993) shows that once a form of sensemaking is (mis)taken as reality, it is enacted as such. The fire-fighters had assumed that the fire was smaller and further away than it was, expecting what was routinely called a 10 o’clock fire (i.e. easily containable by next morning). Misled by this false ‘image’, the smoke-jumpers activity was more relaxed than it should have been. Precious time was wasted and cues of danger inconsistent with this image were missed or rationalised into a pattern that obscured what was actually happening. When the leader finally saw the size of the fire that had in fact already crossed the Gulch just 200 yards in front of them, things happened so fast that personal and collective sensemaking had no time to re-pattern into a more adequate form. Most were dead in a matter of minutes.

Weick describes another swathe of issues as to do with ‘structure’, including the role system of the fire-fighters qua organisation. The fire-fighting team were a ‘minimal organisation’ the activities of which were coordinated by means of established and interlocking roles (namely *crewmembers*, *leader*, and a *deputy*). Weick’s point is that this ‘minimal organisation’ underwent an ‘interactive disintegration of role structure’ in the course of the crisis. Initially, while he finished his meal, the leader had the deputy lead the crew in single file down towards a river, as this location made sense as the safest option for a 10 o’clock fire. After catching up and moving ahead, it was the leader who first saw the actual state of the fire and rapidly abandoned as deadly the initial downwards course towards the river. He gestured to the crew (who were not yet in a position to see the fire) to follow as he quickly turned around, ascended the slope, and began making an escape fire (burning a small patch of land in which to lie low in the hope that the main fire passes quickly over without fuel in that patch). Although following emergency procedure, the leader had no time to properly communicate his sensemaking about this changed plan. In Weick’s account the crew, some still harbouring the false image of a containable fire, ignore the instruction. Amidst the growing cacophony, they fail to understand why the leader is telling them to drop their tools, setting fire to the ground in front of him, and yelling ‘join me’. He is simply thought to have ‘gone nuts’, and the deputy is reported to have said ‘to hell with that, I’m getting out of here’. Hence Weick argues that the ‘minimal organisation’ disintegrated, leaving a meaningless void of ‘everyone to themselves’.

The fact that the leader survived thanks to the escape fire (and all but two of the rest perished) suggests that it might have been better had the authority structure remained intact. Even if the group did not entirely disintegrate, it certainly *changed its form of process* and was no longer a functional organisation capable of coordinated conduct steered towards a shared aim. Weick does not describe this collapse of order as a spontaneous liminal occasion, but his search for a vocabulary of ‘fundamental surprises’ (Reason, 1990), ‘inconceivable’ happenings and ‘incomprehensible’ events (Perrow, 1984) certainly reaches in this direction. He settles on calling the disruption of sensemaking a ‘cosmology episode’: a severe disruption to the coherence and meaning of a cosmology. This foregrounds the *experiences* involved by shifting ‘the analytic focus to feelings and social construction’ (p. 633). It directs attention to the astonishment and terror felt in the face of a *universe* collapsing and ceasing to make sense. Imaginatively invoking the experience of those involved, he describes the collapsed order not as a *déjà vu* (I have seen what is happening before) but a *vu jàdé* (this cannot be happening and I cannot be here). Here Weick invokes Freud’s (1959, p. 28) discussion of panic in military situations as ‘a gigantic and senseless fear’ set free when the ‘mutual ties’ within military groups disintegrate under pressure. With groups whose very survival depends upon coordinated team work, such structural disintegration (and associated transformation of sociality into a ‘free-for-all’) generates a heightened sense of imminent extreme danger. In fact the two surviving crew members had formed a tight diadic unit of mutual support.

### The El Faro Hurricane Disaster

The 790 ft container ship *El Faro* sank in 2015 after sailing into the eye of Hurricane Joaquin, drowning all 33 crewmembers (Weick, 2022). What Weick calls his ‘second-order sensemaking’ was in this case based on published reports by Slade (2018), Foy (2018), Frump (2018), Langewiesche (2018), and Korten (2018). This more recent example is of particular interest because it shows the movement of Weick’s approach towards a form of process thought. Certain basic theoretical ingredients nevertheless remain in place. Amongst all available information, a limited set of ‘cues’ is extracted and assembled by retrospective sensemaking into meaningfully coherent patterns. These patterns are then used to enact a more orderly environment, which in turn yields further cues via cycles of interpretation and action. Problems arise when this patterned sensemaking hardens into an *Idée fixe* such as Captain Davidson’s insistence that the third grade hurricane conditions actually facing the *El Faro* were nothing beyond a typical stormy day in Alaskan seas. Hence for Weick, it was ‘sense that sank them’.

When the *El Faro* left Jacksonville, Florida on the evening of September 29th Davidson was operating with an outdated weather forecast, and with prior experience of having made the personally costly error of changing to a longer route to evade a hurricane that never materialised. He enacted this sensemaking by deciding to stick to the shorter route to San Juan, Puerto Rico, wrongly believing the fast growing hurricane (which he never named as such, preferring ‘disturbance’ or ‘low’ or ‘storm’) was more than 20 hours away. The hurricane was named ‘Joachim’ the following morning (8.00 am on September 30th), and around 20.00 Davidson responded with a slight modification of the route before retiring from the Bridge to sleep. By 23.00 Joachim had grown to category 3 and by the forecast *El Faro* would be 20 miles from its centre and 40 miles from its eye by 4.00am next morning. Around 23.00 officers woke Davidson with this news but the decision was to hold course. Around 12.45am a Second Mate worked out a less dangerous route to avoid their current trajectory to the hurricane’s eye, and notified Davidson around 1.20am. Davidson judges that they will merely touch upon the less dangerous west side of Joachim and should stay on course.

Around 2.15 the engine slows and a little later the officers note the surprising absence of the Captain, who returns to the bridge at 4.09am saying he slept like a baby and that nothing was wrong, no rolling or pitching beyond a typical day in Alaska. Chief mate Schultz agrees but notes an unusual ‘ride’ to the ship (the third deck of which was actually filling with water, making it pitch), which Davidson explains in terms of Alaskan-like gusts. At 4.12 Schults observes a tilt to starboard which Davidson attributes to wind against the containers. Finally at 4.22 Davidson notes conditions are getting worse and repeats this at 5.03. At 5.10 Davidson repeats the typical day in Alaska comment but this time as a question not an assertion. Schultz agrees but Jeff Matthias, experienced with the ship, comments that he has ‘never seen it list like this’.

At 5.16 Davidson asks Schultz if Alaskan seas are like this, Schultz responds that typical Alaskan waves are big but, unlike those produced by hurricanes, predictable enough to course through. Davidson thinks they will only meet the south side and so be safe, but a better observer would have noted that the winds blowing into the port side suggest they had entered the hurricane’s northern eyewall. At 5.18 Davidson says ‘It’s only going to get better from her on, we’re on the back side of it’. 5.43 saw the first realisation that water collected on deck 3 is actually causing the list. At 5.59 it is observed that water is pouring through a scuttle from deck 2 into the deck 3 hold. At 6.13 the engine shuts down and silence reigns – the eye is now 25 miles southeast. At 6.31 Davidson notes ‘we have a flooding list and are disabled and adrift’. At 7.08 the second mate announces that they are 48 miles from the closest landing point. At 7.18 the Captain tells the Second Mate to push the ship’s security alert button, an appeal for rescue. At 7.29 ‘abandon ship’ is called and it sinks.

This chronology (based on Weick, 2022) makes clear that multiple cues were repeatedly ignored by the captain (the listing, the wind on the port side) including direct warnings and alternative proposals from fellow officers, in favour of a form of sensemaking the lethally erroneous nature of which became obvious only when it was too late.

### A Critique and Development of Weick’s Most Recent Theoretical Framework

What is new about Weick’s 2022 approach is the broad theoretical framing in terms of ‘process thinking’. Bergson, Dewey, James and Whitehead are all referenced as inspirations, but he defines process thinking using a quotation from Chia (2017, p.594):

… reality is… perpetually fluxing, changing, and undifferentiated. Pattern, order, identity, and coherence are exceptional stabilizations and deemed to emerge as a consequence of human action, interactions, and interventions into the flux of reality….

This definition, whilst provocative, relies upon a contrast between the reality of undifferentiated flux and its ‘stabilisation’ by human sensemaking. This is a common reading of Bergson’s philosophy. It depends upon an anthropocentric conception of *form* (pattern, order, identity, coherence). Form is understood as an imposition of ‘human action’ upon an actual reality of ‘undifferentiated flux’. Of course human cognition and discourse does indeed selectively edit and form ‘reality’ the better to act on or with it. But this does not mean that, excepting human ‘interventions’, reality lacks inherent form and is a mere flux of unpatterned continuity. In fact, all of nature involves a forming of process, and all natural processes are punctuated by discontinuities which, for Whitehead, are not just real, but the very source of actuality (Stenner, 2008).

To grasp why this matters it is helpful to compare Weick’s new processual framing with his older theory: a hybrid of a cognitive information processing approach and the enactment-oriented constructionism of micro-sociology. Here, sensemaking is an inherently retrospective construction of plausible meanings for any ‘discrepant cues’ that interrupt an activity in process (Maitless and Sonnenshein, 2010, Introna, 2018, Holt & Wiedner, 2024). His notion of ‘cues’ arguably betrays a residual cognitivism which also assumes a meaningless and alien world (reduced to ‘information’) to which the cognitive operations of individuals *lend* order. In the cognitive approach, order then becomes misrecognised as *purely* a function of the way information is processed by the cognitive systems of individuals. The paradoxical complexity of co-evolved and co-developed ‘structural coupling’ is reduced to the ‘input’ of just one side of the dialogical process.

To some degree, Weick escaped these assumptions of cognitive science by means of micro-sociology. For ethnomethodologists and conversation analysts, any structuring of cues into patterns is not merely a function of cognitive information processing, but of the conversations of the micro-interaction order. US psychological and sociological social psychologists might consider themselves radically opposed on this question of the source of social order, but they seem to agree that ‘reality’ is a construction imposed upon a world that lacks its own form, meaning, structure, normativity and order. Hence Weick cites the authority of Morgan et al. (1983: 24): individuals do not live in ‘a wider reality’ but create and operate only with ‘images of a wider reality’. For ‘images’ others substitute ‘cognitions’, ‘social representations’ or some other proxy for *constructions*. But should the fact that we must operate with selective and objectified constructions of wider reality be extended to imply that people are somehow *not* ‘living in, and acting out their lives in relation to, a wider reality’? Cognitive psychologists, ethnomethodologists and conversation analysts often become so enamoured of the fundamental insight that reality is an ongoing accomplishment of our sensemaking (whether cognitive, communicative or ‘praxiological’) that they fail to acknowledge the far from negligible part played by ‘wider reality’ in that accomplishment, giving all the credit (and all the blame) to ‘sensemaking’.

In Weick’s old framing the question of consistency or congruence was, as with Festinger, mostly a matter of the congruence between a multiplicity of ‘cues’ and their configuration into a pattern of sensemaking. Certain cues are ‘preferred’ for attention because they fit a pattern, whilst others are ignored or edited out. Incongruous cues accumulate over time until a now unfit pattern is replaced by a more adequate one (but usually ‘too late’). In a liminality-theoretic framework, Monica Greco and I generalise this as ‘pattern shift’ (Greco & Stenner, 2017) and observe that any such shift (which after all is a matter of the *becoming* of a pattern, necessarily involves a liminal phase of greater or lesser importance. In fact, each ‘anomaly’ or ‘incongruity’ can be viewed as a micro-liminal experience, but a threshold must be crossed, before which the tendency is to *reduce* such liminal experience so that the pattern of pivotal experience may continue. When the threshold is crossed, given a system with sufficient complexity, a new liminal transformation may be occasioned (i.e. the shift from pivotal to liminal metastability outlined above).

In Weick’s new theoretical framing he begins to entertain a different vision of the potentially incongruent ingredients in these crisis experiences. Now the chief contrast is not between meaningless nature and its in*form*ation by cognitions or communications, but between nature as continuous flux and the *discontinuous* forms stamped by the abstractions of sensemaking. It is not simply ‘cues’ within sensemaking that can be mutually incongruous, but sensemaking itself, if it ossifies into rigid ‘arrested’ patterns, can become incongruous with fluxing, changing experience of reality. Weick’s new primary distinction between *continuous experience* and *discontinuous understanding* invokes the authority of William James and claims a new vision of sensemaking: ‘In the following analysis I examine sensemaking as an ongoing attempt to reconcile the continuity of experience with the discontinuity of understanding.’ If sensemaking arrests and distorts, this is because it imposes form upon the ‘flow of continuous experience’:

Davidson’s supposition of a “typical” storm substitutes an abstract conceptual order for the current perceptual order of torrential rain, noise from winds, and repeated auto-pilot alarms as the seas push the ship off course. The seas spun within Joaquin’s counterclockwise eyewall, are made less troublesome because Davidson never called them a “hurricane.”

Hence his thesis is that the *continuity* of Joaquin *became incongruent with* the *discontinuous* supposition that they were sailing in a typical Alaskan storm. The inability to reconcile the incongruity of the two is what, for Weick at least, sank the ship. From my perspective, it would be more simple to say that when sensemaking becomes inflexible and dogmatic (as illustrated by the Captain) it risks losing touch with important aspects of the reality it is supposed to gain purchase on. In extending his concept of *what* can be incongruent, Weick’s new theory allows him to approach the problem of the structural coupling between system and environment. But in holding on to the idea that the actual world lacks form of its own, and in insisting that psychosocial activity is only about creating order out of flux, he seems to miss the crucial role of the structural de-couplings at play in liminal experience in crisis situations.

## Conclusion: There’s Nothing so Theoretical as a Good Practice

When Kurt Lewin said that there is nothing so practical as a good theory he was defending the value of theory for social psychologists in a US environment hostile to and ignorant of all but the most narrow definition of that activity. I find it thought-provoking to reverse his formulation because actually our practice should be guided by the adequate sensemaking provided by good theory, even if for the most part it is not. But the reversal also suggests the value of looking closely at our practice as a source for better theory: our practice, if like Weick we dwell on it, contains rich multitudes that otherwise go unnoticed, and is a treasure trove for theory.

Weick’s work is exemplary in this respect. Although Weick does not spell it out in this way, he makes clear how Captain Davidson’s prior costly experience of seeking to avoid a hurricane that never materialised primed him to want to avoid one of two ‘pathological’ sensemaking-based responses to crises, namely *catastrophising.* Catastrophising is a form of sensemaking that overplays the danger in a situation and in so doing creates a bad outcome (e.g. losing money for the shipping company) that would not have happened but for the catastrophising (a self-fulfilling prophesy). It seems likely that Davidson’s repetition that the circumstances were akin to a typical stormy day at sea in Alaska served, both for him and for those to whom he said it, to inoculate, as it were, from catastrophising. Sadly this seems to have immunised his good sense and led him into the other main pathological response to crisis: denial. Of course, the concept of denial assumes that there actually is a crisis, whilst the concept of catastrophising assumes that there actually isn’t. But it is the nature of these situations to be ambiguously indeterminate and hence to require a judgement call.

Returning briefly to aesthetics and the role of culture and art in making sense of our nature and place, I cannot but think of poor old Oedipus when Weick warns that the actions and decisions of those involved in crises are ‘determinants of the conditions they want to prevent’ (p. 316). Oedipus put out his eyes because, looking only outwards, he could not see that it was he himself who brought about his own downfall. It is one of the functions of ‘tragedy’ to invoke for the future this ‘benefit of hindsight’. Hamlet saw enough to know that he must pause, hesitate, await the right moment. K.V. Shetty, the superintendent for the fateful shift at Bhopal was an administrator with no technical no-how and, according to interviewed workers, was on the verge of panic asking others what should be done. If people in crises tend to attend selectively to things they feel they have capacity to change (and to ignore all else beyond their ken), then at least Shetty had a keen sense of his own inability to manage the situation.

Our sensemaking today is a key contributor to the fact that our social systems are collapsing in crisis all around us and we are being ‘led’ by those with ever more brutalised psychic systems at their disposal, and hence ever more destructive sensemaking is gaining ground. I have probed how liminality theory can help us grasp the relevance of sensemaking to crisis. I have proposed developing common notions of upset equilibrium and homeostasis into a contrast between pivotal and liminal metastability, and I have traced its relevance through some key figures in psychology. This calls attention to the delicate balance of liminal metastability arising when taken for granted structural couplings are subject to disruption. It also requires a process ontology capable of accommodating the complex and multi-levelled nature of every aspect of life. From this perspective liminal experience and sensemaking is not the privilege of human beings but applies ‘all the way down’. Form is not just a human imposition, but a feature of all forms of process.

It would be an error, however, to overstate the power of thought in our lives. We are immersed in action: we occasionally think, and occasionally our ideas get purchase on our action. At best we can take note when an idea seems to have some purchase in one of the many spheres of experience in which we move. When that is the case, it is wise to cherish that idea. Our actions, meanwhile, will keep running on ahead of us.

## Data Availability

No datasets were generated or analysed during the current study.
